# Uterine sarcoma with KAT6B/A::KANSL1 fusion: a molecular and clinicopathological study on 9 cases

**DOI:** 10.1007/s00428-024-03994-3

**Published:** 2024-12-04

**Authors:** Pavel Dundr, Jiří Dvořák, Michaela Krausová, Jan Hojný, Nikola Hájková, Ivana Stružinská, Kristýna Němejcová, Ondřej Ondič, Michael Michal, Květoslava Michalová, Alberto Berjón, Marcin Jedryka, Mariusz Książek, Tymoteusz Poprawski, Janusz Ryś, Nataliya Volodko, Ignacio Zapardiel, Tomáš Zima, David Cibula, Renata Poncová, Radoslav Matěj, Michaela Kendall Bártů

**Affiliations:** 1 Department of Pathology, First Faculty of Medicine, Charles University and General University Hospital in Prague, Studničkova 2, Prague 2, 12800 Czech Republic; 2 Department of Pathology, Charles University, Faculty of Medicine in Plzeň, Bioptical Laboratory, Ltd, Plzeň, Czech Republic; 3 Pathology Department, La Paz University Hospital, Madrid, Spain; 4 Oncology Department, Wroclaw Medical University, Wroclaw, Poland; 5 Oncological Gynecology Department, Lower Silesian Oncology, Pulmonology and Hematology Center, Wroclaw, Poland; 6 Department of Tumor Pathology, Maria Sklodowska-Curie National Research Institute of Oncology, Krakow Branch, Krakow, Poland; 7 Danylo Halytsky Lviv National Medical University, Lviv, Ukraine; 8 Gynecologic Oncology Unit, La Paz University Hospital, Madrid, Spain; 9 Institute of Medical Biochemistry and Laboratory Diagnostics, First Faculty of Medicine, Charles University and General University Hospital in Prague, Prague, Czech Republic; 10 Gynecologic Oncology Centre, Department of Gynaecology, Obstetrics and Neonatology, First Faculty of Medicine, Charles University and General University Hospital in Prague, Prague, Czech Republic; 11 Department of Pathology, Charles University, 3rd Faculty of Medicine, University Hospital Kralovske Vinohrady, 10034 Prague, Czech Republic; 12 Department of Pathology and Molecular Medicine, Third Faculty of Medicine, Charles University, Thomayer University Hospital, Prague, Czech Republic

**Keywords:** Uterine tumor, Endometrial stromal sarcoma, Next generation sequencing, KAT6B/A::KANSL1 fusion

## Abstract

Uterine sarcomas with *KAT6B/A::KANSL1* fusion represent a new entity characterized by bland morphology, commonly with hybrid features of low-grade endometrial stromal sarcoma (LG-ESS) and tumors with smooth muscle differentiation. In our study, we performed a detailed morphological, immunohistochemical, and molecular analysis of 9 cases of these tumors. Six of those had been originally diagnosed as LG-ESS, one as leiomyoma, one as leiomyosarcoma, and the remaining case as sarcoma with the *KAT6B/A::KANSL1* fusion. Seven cases showed overlapping features between endometrial stromal and smooth muscle tumors, one case resembled cellular leiomyoma, and one case resembled high-grade endometrial stromal sarcoma. Immunohistochemically, the tumors showed a common expression of smooth muscle markers and endometrial stromal markers. Molecular findings showed the *KAT6B/A::KANSL1* fusion in all cases (by NGS and FISH). In addition, mutations affecting genes such as *TP53*, *PDGFRB*, *NF1*, *RB1*, *PTEN*, *ATM*, *RB1, FANCD2,* and *TSC1* were present in all 5 cases with aggressive behavior. One patient with no evidence of disease showed no additional mutations, while another harbored a mutation of a single gene (*ERCC3*). Of the 8 patients with available follow-up, two died of disease, 3 are currently alive with disease, and 3 have no evidence of disease. The correct recognition of tumors with the *KAT6B/A::KANSL1* fusion is essential because despite the bland morphological features of most cases, these tumors have a propensity for aggressive behavior.

**Supplementary Information:**

The online version contains supplementary material available at 10.1007/s00428-024-03994-3.

## Introduction

The landscape of uterine mesenchymal tumors is changing, especially due to the increased understanding of their molecular features [1–4]. The diagnosis of these tumors is generally based on their morphological and immunohistochemical features; however, in some cases, it cannot be achieved with certainty without molecular testing. This is especially important for tumors showing equivocal or overlapping features between defined categories, such as those with features between low-grade endometrial stromal sarcoma (LG-ESS) and smooth muscle tumors, inflammatory myofibroblastic tumors, PEComas, myxoid uterine tumors, tumors suggestive of fusion-positive high-grade endometrial stromal sarcoma (HG-ESS), and some others [1, 5–7]. Previous studies have shown that the diagnostic agreement between some tumors is rather poor and molecular testing can be helpful in increasing diagnostic agreement and reproducibility of established entities [2, 8]. Moreover, there are some new entities in which molecular testing is needed for the correct diagnosis. This includes sarcomas with *KAT6B/A::KANSL1* fusion, described in 2022 by Agaimy et al. in a study on 13 cases [9]. Recently, the cases from this study together with 5 additional new cases were analyzed in a new work focusing on methylation profiling of these tumors [10]. Since they were originally described, another study reported a series of 12 patients with tumors classified as sarcomas with *KAT6B/A::KANSL1* fusion [11]. Additionally, three cases of smooth muscle tumors with *KAT6B::KANSL1* fusion were described in the literature, two of which were diagnosed as leiomyomas and one as a leiomyosarcoma [12–14]. According to the available data, most of these tumors have overlapping features with tumors with endometrial stromal and smooth muscle differentiation. Majority of the described cases have low-grade features mostly overlapping with endometrial stromal and smooth muscle differentiation. Some of the tumors resemble the fibroblastic/fibromyxoid variant of ESN/LG-ESS, while others are more similar to cellular leiomyoma. In general, the morphological and immunohistochemical features typically vary between those of ESN/LG-ESS and smooth muscle tumors and reliably rendering an accurate diagnosis without molecular testing is currently impossible. Minority of cases can show high-grade features, and these cases resemble high-grade endometrial stromal sarcoma or undifferentiated uterine sarcoma. The correct diagnosis of sarcoma with *KAT6B/A::KANSL1* fusion is of utmost importance, given that these tumors tend to behave aggressively even in cases which exhibit deceptively bland morphology. According to previously published cases and our results, about 14% of patients died of disease and 20% is alive with disease. In this study, we collected 9 uterine sarcomas with the *KAT6B/A::KANSL1* fusion and analyzed their morphological features, together with a comprehensive analysis using immunohistochemistry, NGS (DNA and RNA), and FISH.

## Material and methods

Nine cases were available for analyses, one of which was from the consultation files of one participant (MM). The remaining eight cases were detected among a group of 379 uterine mesenchymal tumors representing a series of molecularly analyzed cases in the rare gynecologic sarcoma (REGYS) project. Briefly, in this project, 379 tumors initially diagnosed as LG-ESS, HG-ESS, undifferentiated uterine sarcoma, or myxoid leiomyosarcoma were collected and extensively analyzed, including the assessment of morphological, immunohistochemical, and molecular features. Among these tumors, 8 cases with *KAT6B/A::KANSL1* fusion were identified, and these tumors are in detail described in this current study, together with one case from the consultation files of one participant of the study (in which the molecular testing was initially a part of the diagnostic work-up). The details concerning the REGYS project are described in other study (manuscript under review). In two of these cases, material from the recurrent tumor was also available.

### Immunohistochemical analysis

Immunohistochemistry was performed using 4-μm-thick sections of formalin-fixed and paraffin-embedded (FFPE) tissue partly on tissue microarrays and partly on whole tissue sections [9]. TMA was used for cases from the REGYS project. The only exception was case #3 (which tissue was lost in the TMA) and two additional cases (#8 and #9), where whole tissue sections were used instead. For construction of the TMAs, eligible areas of each tumor were identified, and two tissue cores (each 2.0 mm in diameter) were taken from the donor block, using the tissue microarray instrument TMA Master (3DHISTECH Ltd., Budapest, Hungary). The antibodies used included estrogen receptor, progesterone receptor, smooth muscle actin, desmin, caldesmon, calponin, CD10, IFITM1, transgelin, smoothelin, BCOR, BCORL1, WT1, p53, cyclin D1, and MMR proteins (MLH1, PMS2, MSH2, and MSH6). The clones, manufacturers, dilutions, and staining instruments for all of the antibodies are summarized in Supplementary Table 1. The immunohistochemical results were assessed semiquantitatively according to the overall percentage of positive cells (0–100%) and then also using the *H*-score method (based on the assessment of the percentage of positive cells and the level of staining intensity: weak 1 + , moderate 2 + , and strong 3 +). The final *H*-score for each case was calculated by adding the multiplications of the different staining intensities according to the following formula: 1x (% of cells 1 +) + 2x (% of cells 2 +) + 3x (% of cells 3 +), resulting in an *H*-score value of 0–300. The cases were classified based on the overall percentage of positive cells as negative (entirely negative or < 5% of positive tumor cells) or positive (5–100% of positive tumor cells). Positivity in 5–49% of tumor cells was considered as “focal,” positivity in ≥ 50% of tumor cells as “diffuse.” For MMR proteins, a 5% cut-off of positive cells was used as the upper limit for loss of expression and the intensity of staining should be equal or stronger compared to internal control. The p53 protein expression was assessed as either the “wild-type” or “aberrant type.” The “aberrant type” of staining was defined as diffuse strong nuclear positivity of at least 80% of tumor cells, cytoplasmic positivity, or the complete absence of staining with positive internal control.

### Molecular analysis

In all cases, the molecular analysis was performed using the FFPE tissue material, from which targeted capture next-generation sequencing (NGS) of DNA (786 genes or gene parts; 2440 kbp of target sequence; 1992 kbp of coding sequence; Supplementary Table 2) and whole transcriptome RNA-Seq of total RNA (rRNA ribodepletion approach) were performed. DNA library preparation was performed using the KAPA HyperPlus kit (Roche) following the KAPA HyperCap Workflow v3.0. For transcriptome RNA-Seq, 1 µg of total RNA (> 200 bp) was subjected to rRNA and globin mRNA depletion using the NEBNext Globin & rRNA Depletion Kit (New England Biolabs). Libraries were constructed using the KAPA RNA HyperPrep Kit (Roche) with the following modifications: RNA fragmentation at 65 °C for 2 min, KAPA UMI adapters at a final concentration of 750 nM, and 13 PCR cycles using KAPA UDI Primer Mixes.

Libraries were pair-end sequenced on either NovaSeq 6000 or NextSeq 500 instruments (Illumina, San Diego, CA, USA) using the NovaSeq 6000 S2 Reagent Kit v1.5 (300 cycles) or NextSeq 500/550 High Output Kit v2.5 (150 cycles), respectively. FastQ files from 300-cycle sequencing were trimmed to match 150-cycle sequencing depth. Transcriptome libraries were sequenced to a target depth of 60 million unique reads per sample.

Bioinformatic analysis of raw FASTQ files was conducted using a custom pipeline implemented in CLC Genomics Workbench v23.0.5 (CLC; QIAGEN) with the GRCh38 human genome build as reference. The comprehensive parameters about pipeline settings have been described in previous work [15]. Detected variants were evaluated as described in Struzinska et al., 2023 [16]. Tumor mutation burden (TMB) was calculated by counting all coding synonymous and non-synonymous variants (with no record in dbSNP or ClinVar database) divided by the total length of the sequenced coding region. Only the coding areas with coverage > 100 × were used for TMB calculation.

Fusions were detected and annotated using CLC module “Detect and Refine Fusion Genes” which is comprised of i) detection of potential fusions by remapping all the unaligned ends of reads longer than 20 bp, ii) creation of sequence templates of the potential fusion genes, and iii) refining the fusions by remapping all reads to these fusion templates. After the bioinformatic analysis, all refined fusion events were confirmed by cDNA qPCR approach.

Exploratory gene expression analysis, including principal component analysis (PCA) and unsupervised hierarchical clustering analysis, was performed using the CLC with Manhattan distance and Complete linkage parameters (max. 10 thousand features; min. 5 thousand counts in at least one sample). Comparative differential gene expression analysis was conducted between the 9 *KAT6B/A::KANSL1* samples and 123 low-grade endometrial stromal sarcoma (LG-ESS) tumor samples. The latter cohort is a part of the rare gynecologic sarcomas (REGYS) study project, which is currently under review. All samples in the LG-ESS comparison group (*n* = 123) underwent rigorous histopathological and immunohistochemical evaluation to confirm the diagnosis of LG-ESS. Furthermore, these samples were comprehensively screened for the presence of known gene fusion events commonly associated with LG-ESS. Specifically, we identified 4 cases harboring an *EPC1::PHF1* fusion, 9 cases with a *JAZF1::PHF1* fusion, 65 cases with a *JAZF1::SUZ12* fusion, and 7 cases containing a *MEAF6::PHF1* fusion. Additional 13 cases displayed other rare fusion events, while 25 cases lacked any detectable gene fusions. Importantly, none of the LG-ESS samples in this cohort demonstrated the *KAT6A/B::KANSL1* fusion which is the focus of this current study. The “Differential Expression in Two Groups” module in GW (multi-factorial statistics based on the negative binomial generalized linear model) was used, where the TMM normalization method is used for transcriptome RNA-Seq data. Only genes with a maximal group average transcript per million (TPM) value above 10 and significant differences (Bonferroni correction) were selected for further evaluation.

GOAT online (version 1.0, http://ftwkoopmans.github.io/goat) was used to perform the gene set enrichment analyses using gene sets from the Gene Ontology database (version gene2go_2024-01–01) [17, 18]. The input gene list contained 3563 genes which were significantly differentially expressed and its effect size-derived gene scores were used to test for enriched gene sets that contained at least 10 and at most 1500 genes (or 50% of the gene list, whichever was smaller) that overlapped with the input gene list. Multiple testing correction was independently applied per gene set “source” (i.e., GO_CC, GO_BP, and GO_MF) using the Bonferroni adjustment and, subsequently, all *p*-values were adjusted (again) using the Bonferroni adjustment to account for 3 separate tests across “sources.” The significance threshold for adjusted *p*-values was set to 0.05. The results were visualized as ridgeline charts using the Express Analyst tool (www.expressanalyst.ca), and the functional protein association networks were analyzed using the STRING Database (https://string-db.org/) [19, 20].

### Fluorescence in situ hybridization

For the detection of *KANSL1, KAT6A*, and *KAT6B* rearrangement, *KAT6B::KANSL1* fusion, *KANSL1* break apart, *KAT6A* break apart, *KAT6B* break apart, and *KAT6B::KANSL1* dual fusion probes were used (HealthCare Biotechnology Ltd. Inc, Wuhan, Hubei, China). The FISH analysis was performed and interpreted as described elsewhere [21].

## Results

Six of the tumors were originally diagnosed as LG-ESS, one as leiomyoma, one as leiomyosarcoma, and the last case (in which molecular testing was a part of the diagnostic work-up) as a sarcoma with the *KAT6B::KANSL1* fusion.

### Clinical findings

The age at the time of diagnosis ranged from 37 to 66 years (average 50.3 years). The follow-up was available for 8 patients. The last patient is a recent case (#9) without follow-up. All patients were primary treated by hysterectomy (7 of those with bilateral adnexectomy). From the 8 patients with follow-up available, aggressive behavior was seen in 5 patients (62.5%). Two patients died of disease (DOD) 99 months and 72 months after the initial diagnosis. The first patient had three intra-abdominal recurrences 7 and 8 years after the diagnosis treated by surgery, but the last one was not eligible for surgical treatment and the patient died few months later (case #7). The second patient developed lung metastases 6 years after the initial diagnosis and died soon after that (case #8). Three patients are currently alive with disease. One of them had two relapses, the first occurring two years after the diagnosis (tumor thrombus in a pelvic vessel), while the second 8 years after the diagnosis (abdominal recurrence), and this patient is currently receiving palliative treatment (case #4). The second patient experienced an abdominal recurrence 7 years after the diagnosis and is currently (25.8 years after the diagnosis) alive with disease (abdominal and pelvic recurrence) (case #5). The third patient developed lung metastasis 9 months after the diagnosis and is currently alive with disease (case #6). Three patients with follow-up durations of 32, 67, and 75 months are without evidence of disease. The clinical data are summarized in Table 1.

**Table 1:** Clinicopathological characteristics of patients

Characteristic\# case	1	2	3	4	5	6	7	8	9	Average/median
Age at diagnosis (years)	37	48	44	54	48	66	60	50	46	**50/48**
BMI	30	34	25	28	21	26	NA	NA	NA	**27/26**
Menopausal status	Pre	Pre	Pre	Post	Pre	Post	NA	NA	NA	
Max. tumor size (mm)	120	30	NA	NA	40	100	50	200	25	**80/50**
FIGO	IB	IA	IA	IA	IIA	IB	NA	NA	NA	
LVSI	No	No	No	No	Yes	Yes	No	NA	NA	
Type of surgery	HYE, AE	HYE, AE	HYE, AE	HYE, AE	HYE, AE	HYE	HYE, AE	HYE, AE	HYE	
Adjuvant treatment	No	No	No	NA	CHT/RT	CHT	NA	NA	NA	
Distant metastasis	No	No	No	NA	No	Yes	NA	Yes	NA	
Follow-up (months)	67	32	75	102	310	9	99	72	0	**83/70**
Disease status	NED	NED	NED	AWD	AWD	AWD	DOD	DOD	NA	

*AE* Adnexectomy, *AWD* alive with disease, *CHT* chemotherapy, *DOD* died of disease, *HYE* hysterectomy, *NA* not available, *NED* no evidence of disease, *RT* radiation therapy

### Pathological findings

**Fig. 1:**
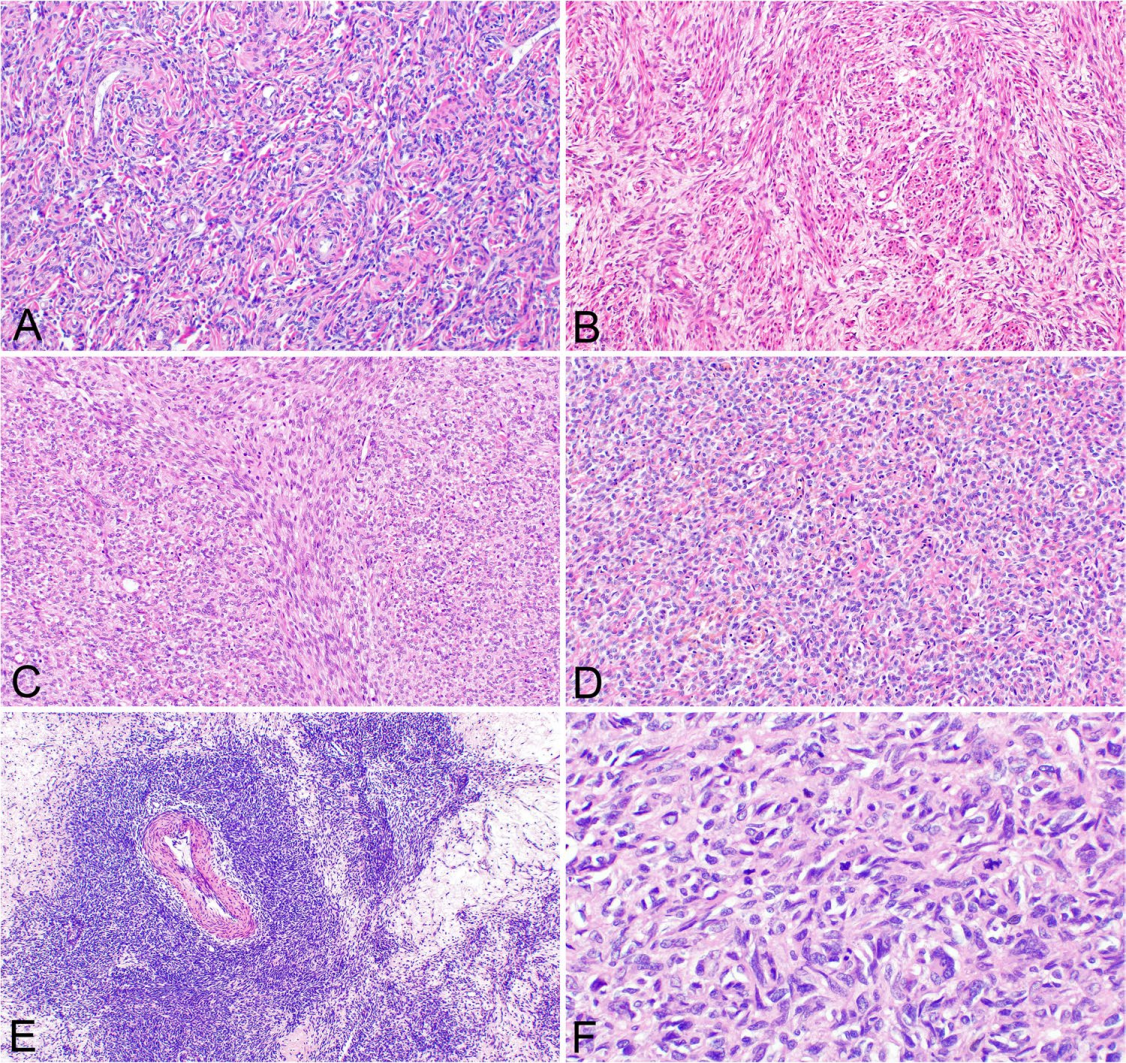
Histological features of *KAT6B/A::KANSL1* fused sarcomas. LG-ESS-like features with typical spiral arterioles (**A**) (case #1; 200x). Tumor with hybrid fibroblastic and smooth-muscle features (**B**) (case #9; 200x). Nonspecific spindle cell (**C**) and round cell (**D**) morphology with overlapping features between smooth muscle and endometrial stromal differentiation (case #4 and #7; 200x). Tumor resembling cellular leiomyoma with regressive changes (**E**) (case #3; 100x). Tumor with high-grade features (**F**) (case #6; 400x)

All tumors were located in the uterine corpus. The information about size was available in 7/9 primary cases. The maximum dimension ranged from 25 to 200 mm (average 80.7 mm). Two tumors were sharply demarcated, two tumors showed infiltrative growth, and in 5 tumors the information about margin is not available. Histologically, 7 tumors showed low-grade features with a variable proportion of areas resembling the fibroblastic variant of ESN/LG-ESS, areas with equivocal hybrid features between endometrial stromal differentiation (mostly with fibroblastic or fibromyxoid features) and smooth muscle features (Fig. 1A–D). These tumors mostly consisted of cells with mild nuclear atypia, and only three cases showed a moderate degree of atypia. Mitoses ranged from 0 to 12/10 HPF (average 6), focal tumor necrosis was present in one of these cases. The tumor cells were spindle or oval. Focal hyalinization was present in 3 tumors. Focal myxoid areas were present in three tumors, and one tumor showed pure myxoid morphology in the lung metastasis (case #8). In another case (case #4), in which material from the metastasis was available, the primary tumor showed predominant smooth muscle-like morphology, while the metastasis (intravascular tumor thrombus) presented with hybrid LG-ESS and smooth muscle features. All but two tumors (#5 and #6) showed small arterioles typically occurring in LG-ESS, and all but one tumor (#6) showed thick-walled vessels. The hemangiopericytoma-like vessels were present in only 2 tumors (#2 and #5).

Two of the tumors showed different features, one of which (#3) presented with features resembling cellular leiomyoma with regressive changes, including geographical fibrosis and focal necrosis (Fig. 1E). Focally, areas with sex cord-like features (< 5%) were also present in this tumor. Tumor cells were spindled with mild nuclear atypia, and no mitoses were found. The other tumor (#6) showed high-grade features with oval and spindle cells, necroses, and mitoses up to 37/10 HPF (Fig. 1F).

### Immunohistochemical findings

**Fig. 2:**
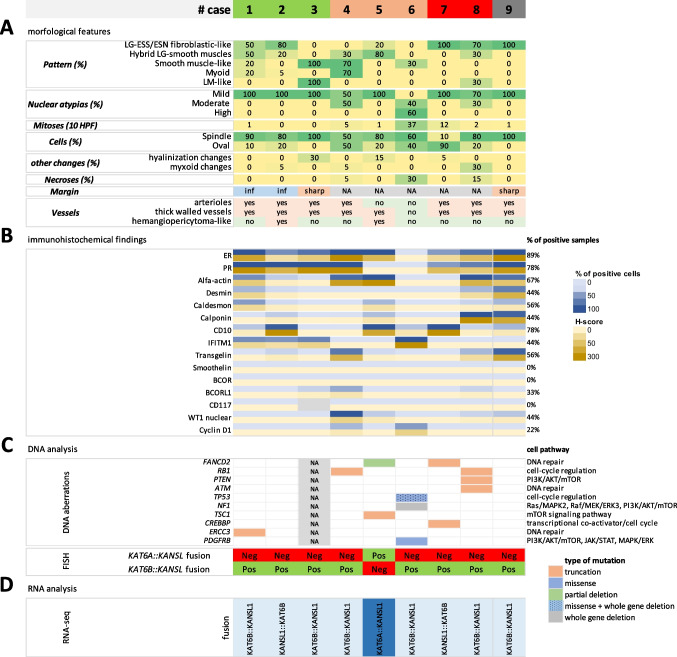
Comprehensive characterization of nine sarcomas harboring KAT6B/A::KANSL1 fusion. Summary of morphological features observed (**A**). Results of immunohistochemical staining (**B**). Molecular landscape depicting genomic alterations and *KAT6A/B::KANSL1* fusion characterization across the analysed samples (**C**, **D**). Legend: AWD: alive with disease; DOD: died of disease; inf: infiltrative; NA: not available; NED: no evidence of disease; Neg: negative; Pos: positive. Color-coded genetic alterations: orange, truncating variants; blue, missense variants; gray, copy number deletions; green, large insertions/deletions; blue + gray, concurrent missense variants and copy number deletions

The immunohistochemical profile was nonspecific. Most tumors showed expression of estrogen receptor (8/9; 88.9%) and progesterone receptor (7/9; 77.8%). The expression of smooth muscle markers was variable and frequently only focal. Of these, smooth muscle actin was the most commonly expressed marker (6/9; 66.7%) with diffuse positivity in 5/9 (55.6%) cases, followed by transgelin, caldesmon, and calponin (each 5/9, 55.6%; diffuse positivity in 2/9 (22.2%), 1/9 (11.1%), and 2/9 (22.2%) cases, respectively), and desmin (4/9; 44.4%, diffuse positivity in 1/9 (11.1%) cases). Only 3 tumors showed co-expression of all these markers, but it was usually only focal (5–30% of tumor cells). No case showed expression of smoothelin. Concerning the endometrial stromal markers, CD10 was expressed in 7/9 (77.8%, diffuse positivity in 3/9 (33.3%) cases) and IFITM1 in 4/9 (44.4%; all cases diffuse positivity) of the tumors. WT1 was expressed in 4/9 (44.4%) of the tumors, but only one case showed diffuse expression, and in all cases the expression was weak. Cyclin D1 was focally weakly positive in 2/9 (22.2%) of cases. All tumors showed wild-type expression of p53, including one tumor (case #6) in which the *TP53* mutation was found. All tumors had retained expression of MMR proteins. The immunohistochemical findings including the extent of expression and H-score are summarized in Fig. 2 and Table 2.

**Table 2:** Summary of immunohistochemical findings

IHC marker		Case 1	Case 2	Case 3	Case 4	Case 5	Case 6	Case** 7**	Case 8	Case 9	Median (IQR)	Mean (SD)
**ER**	Overall positivity (%)	100	60	70	100	100	0	50	80	100	80 (60, 100)	73.3 (33.5)
	*H*-score	235	60	100	300	100	0	50	150	280	100 (60, 235)	142 (107.0)
**PR**	Overall positivity (%)	100	100	90	100	0	0	50	70	100	90 (50, 100)	68 (42.1)
	*H*-score	300	185	250	300	0	0	110	120	250	185 (110, 250)	168 (117.6)
**Alfa-aktin**	Overall positivity (%)	60	15	3	85	100	0	0	100	80	60 (3, 85)	49 (95.6)
	*H*-score	120	15	3	255	170	0	0	180	130	120 (3, 170)	97 (95.6)
**Desmin**	Overall positivity (%)	30	5	0	0	0	0	0	30	70	0 (0, 30)	15 (24.2)
	*H*-score	90	15	0	0	0	0	0	60	180	0 (0, 60)	38 (62.3)
**Caldesmon**	Overall positivity (%)	50	5	0	0	30	0	0	10	30	5 (0, 30)	14 (18.3)
	*H*-score	75	15	0	0	30	0	0	10	30	10 (0, 30)	18 (24.8)
**Calponin**	Overall positivity (%)	0	15	0	1	25	0	0	100	90	1 (0, 25)	26 (40.3)
	*H*-score	0	20	0	2	26	0	0	280	210	2 (0, 26)	60 (106.9)
**CD10**	Overall positivity (%)	20	95	0	0	100	20	100	10	30	20 (10, 95)	42 (43.6)
	*H*-score	20	285	0	0	130	23	200	10	30	23 (10, 130)	78 (43.6)
**IFITM1**	Overall positivity (%)	60	55	60	0	0	90	0	0	0	0 (0, 60)	29 (36.3)
	*H*-score	80	55	80	0	0	180	0	0	0	0 (0, 80)	44 (62.1)
**Transgelin**	Overall positivity (%)	0	10	0	75	5	0	0	30	90	0 (0, 30)	23 (35.1)
	*H*-score	0	20	0	190	5	0	0	60	210	5 (0, 60)	54 (85.2)
**Smoothelin**	Overall positivity (%)	0	0	0	0	0	0	0	1	0	0 (0, 0)	0.1 (0.3)
	*H*-score	0	0	0	0	0	0	0	1	0	0 (0, 0)	0.1 (0.3)
**BCOR**	Overall positivity (%)	0	0	0	0	0	0	0	0	0	0 (0, 0)	0 (0)
	*H*-score	0	0	0	0	0	0	0	0	0	0 (0, 0)	0 (0)
**BCORL1**	Overall positivity (%)	0	0	10	40	0	0	0	10	0	0 (0, 10)	7 (13.2)
	*H*-score	0	0	10	40	0	0	0	10	0	0 (0, 10)	7 (13.2)
**WT1 nuclear**	Overall positivity (%)	0	0	0	99	15	0	0	10	20	0 (0, 15)	16 (32.1)
	*H*-score	0	0	0	99	15	0	0	10	20	0 (0, 15)	16 (32.1)
**Cyclin D1**	Overall positivity (%)	0	0	0	30	0	45	0	0	0	0 (0, 0)	8 (16.9)
	*H*-score	0	0	0	30	0	60	0	0	0	0 (0, 0)	10 (21.2)

Values are shown as median (*IQR* interquartile range presented as Q1, Q3) and mean (*SD* standard deviation) for each marker

### Molecular findings

RNA sequencing showed 8 cases with the *KAT6B::KANSL1* fusion (*KAT6B* (NM_012330.4): r.1_1135 *KANSL1* (NM_001193466.2): r.2995_5331) and one case (#5) with the *KAT6A::KANSL1* fusion (*KAT6A* (NM_006766.5): r.1_1012 *KANSL1* (NM_001193466.2): r.2995_5331). The analysis confirmed that all the detected *KAT6B/A::KANSL1* fusions maintained the reading frame. The precise genomic coordinates of the fusion breakpoints are provided in Supplementary Table 3. DNA sequencing was available in 8/9 cases and the detected mutations are summarized in Fig. 2 and in detail in Supplementary Table 4.

Briefly, two of these cases (#2 and #9) did not harbor any mutations—one was with no evidence of disease at the last follow up (#2), while the other is without available follow-up (#9). Another patient with no evidence of disease showed a pathogenic mutation in a single gene (*ERCC3*; c.1762dupG, p.(Glu588fs)). Of the patients with aggressive behavior, one case showed a known pathogenic mutation in *TP53* (c.524G > A, p.(Arg175His)) combined with a deletion spanning *the TP53* gene, *PDGFRB* (c.1998C > A, p.(Asn666Lys)), and a deletion of *NF1* (case #6). One case showed a single truncating mutation (*RB1*; c.1363C > T, p.(Arg455Ter)) (case #4), with another case showing a mutation in *PTEN* (c.1040del, p.( Phe347fs)), *ATM* (c.1402_1403del, p.( Lys468fs))*,* and *RB1* (c.157del, p.( Glu53fs)) (case #8). Two cases showed two concurrent mutations—case #5 harbored *FANCD2* deletion and *TSC1* (c.3351_3352insC, p.(Lys1118fs)); and case #7 harbored mutations in *FANCD2* (c.1632_1633del, p.(Asn545fs) and *CREBBP* c.776dup, p.(Gln260fs)). All samples were microsatellite stable. Based on our analysis, all tumors had a low mutational burden (TMB min = 0.5; max = 4.1; median = 2.3 mut/Mb.).

**Fig. 3:**
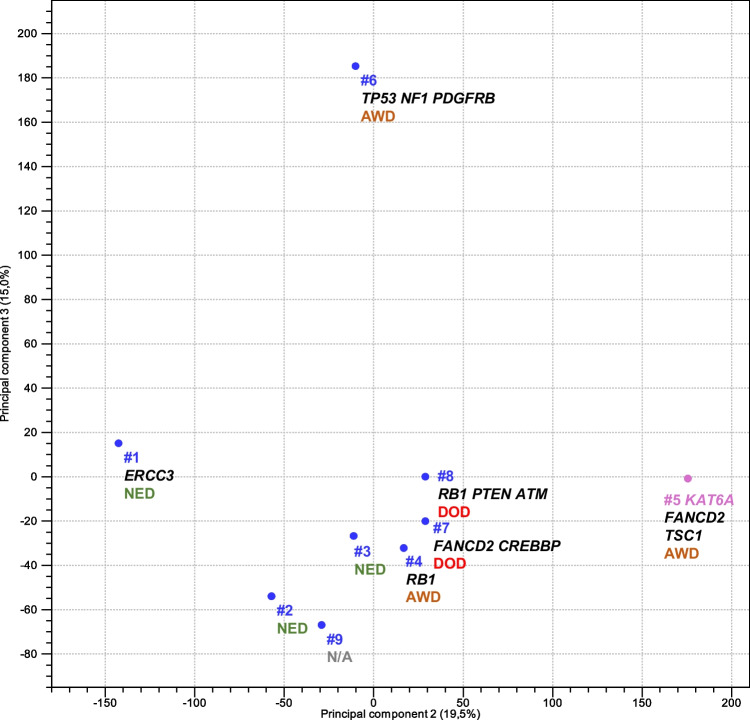
A principal component analysis (PCA) plot depicting the second and third principal components. The plot differentiates the *KAT6A* and *KAT6B* fusion partners through distinct color coding. Individual gene aberrations are denoted by their corresponding gene symbols. Disease status at last follow-up control is indicated for each case (AWD: alive with disease, DOD: died of disease, and NED: no evidence of disease)

An exploratory gene expression analysis was conducted to elucidate the transcriptomic patterns within our *KAT6B/A::KANSL1* cohort. The principal component analysis (PCA) and unsupervised hierarchical clustering methodologies were employed, revealing distinct gene expression profiles between specimens harboring *KAT6B* fusion partners and the sample exhibiting a *KAT6A* gene fusion partner (#5). Furthermore, case 6, characterized by DNA aberrations in *TP53*, *NF1*, and *PDGFRB*, demonstrated a divergent gene expression pattern, clustering independently from the other samples. These findings are visually represented in the PCA plot (Fig. 3) and in Supplementary Fig. 1.

Differential gene expression analysis was conducted to compare the *KAT6B/A::KANSL1* sample group with the LG-ESS group. Among the most upregulated genes in the *KAT6B/A::KANSL1* cohort were *CHAF1B, TARBP1, RALGPS1, LYSMD4, FAM53A, PPFIA2, SH3GL3,* and *SLITRK4*. Conversely, the most downregulated genes included *GASK1B, SULT1C4, IP6K2, ALPK1, SRSF2, MR1, ZBTB38*, and *EFCAB2*. To visualize the differential gene expression patterns, violin plots were generated, with particular emphasis on case 6 (Supplementary Fig. 2). Moreover, we included a PCA analysis depicting the global transcriptional profiles of these two groups, which is provided in Supplementary Fig. 3. We also conducted an intragroup analysis of gene expression within the *KAT6B/A::KANSL1* cohort, stratified by clinical outcome. Our findings revealed different expression patterns for *SLC2A12* and *ABCA8* genes between the adverse outcome group (AWD/DOD; cases #4–8) and the favorable outcome group (NED; cases #1–3). These results are summarized in Supplementary Fig. 4 and in Supplementary Table 5.

**Fig. 4:**
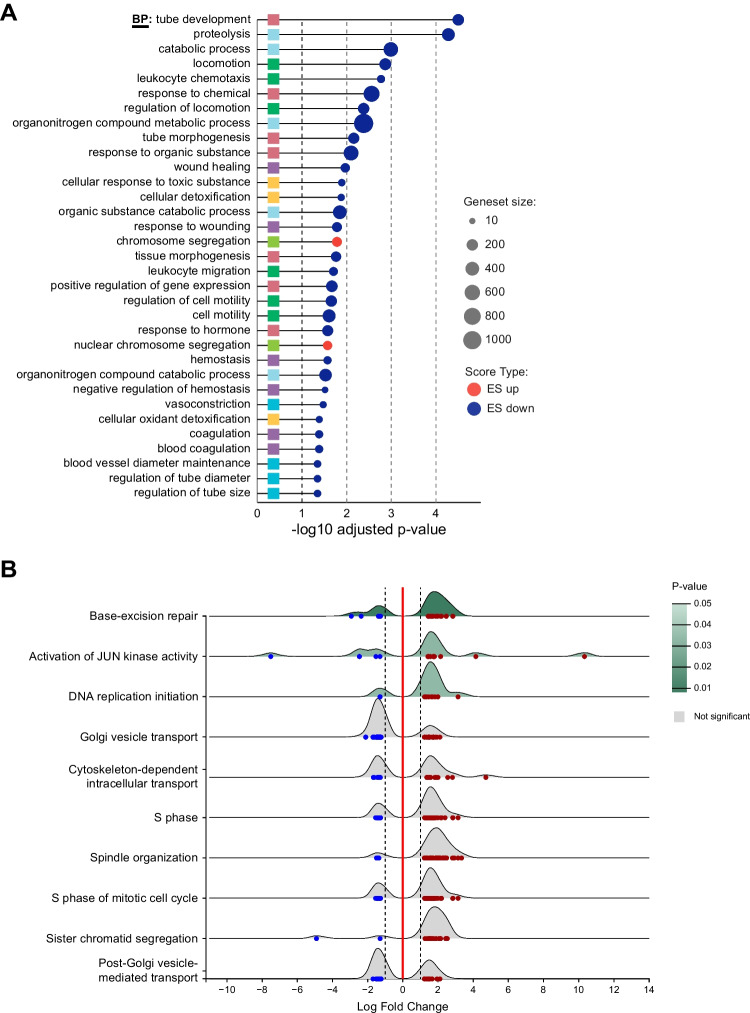
Gene set enrichment analyses of genes differentially expressed in *KAT6B/A::KANSL1* sarcomas when compared to LG-ESS. Gene set ordinal association test (GOAT) was used to identify enriched categories within the GO database domain biological process (BP) (**A**). The cellular component (CC) and molecular functions (MF) domains can be found in Supplementary Fig. 5 (A, B). Effect size (ES) score type “ES up” (red circles) indicates that the gene set is enriched in genes with a positive gene effect size, “ES down” (in dark blue) indicates enrichment of negative gene effect sizes. Colored squares indicate hierarchical clustering of the gene sets. GSEA results visualized as a ridgeline plot, with overrepresentation analysis performed against the Gene Ontology (GO) biological process domain (**B**). Blue and red dots indicate expression change in individual genes. The underlying data accompanying both figures are provided in Supplementary Table 6 and 7

Gene set enrichment analysis (GSEA) of the transcriptome of tumors carrying the* KAT6B/A::KANSL1* fusion versus the LG-ESS tumors highlighted an overrepresentation of genes involved in cell cycle progression. Specifically, we recorded upregulation of numerous genes that participate in DNA replication such as DNA unwinding enzymes (e.g., *DDX11*, *MCM2-6*, and *MSM8*) and the catalytic subunits or cofactors of DNA polymerase (e.g., *POLA*, *POLD*, and *PCNA*, respectively), or proteins implicated in replication-associated proofreading mechanisms (*POLE*) (Fig. 4 and Supplementary Table 6). Conversely, the category of downregulated genes displayed enrichment for genes that are responsible for ubiquitin-dependent proteasomal degradation (Supplementary Fig. 5 and Supplementary Table 7).

### Fluorescence in situ hybridization

The FISH analysis detected rearrangements/fusions of the *KAT6B and KANSL1* genes in 8/9 examined tumors. In one case harboring the *KAT6A::KANSL1* fusion, the breaks of *KAT6A* and *KANSL1* were detected and accompanied by negative *KAT6B::KANSL1* fusion probe result. The results are summarized in Fig. 2.

## Discussion

Sarcomas with *KAT6B/A::KANSL1* fusion were first described in one retroperitoneal and one uterine tumor originally diagnosed as leiomyoma, followed shortly after by a tumor initially diagnosed as uterine leiomyosarcoma [12–14]. However, the chromosomal rearrangements at specific loci on chromosome 10q and 17q had been sporadically reported in smooth muscle tumors before [22–24]. In 2022, Agaimy et al. described a series of 13 uterine tumors with *KAT6B/A::KANSL1* fusion and suggested, for the first time, that these cases represent a distinct entity [9]. In 2023, Trecourt et al. reported a series of further 12 patients [11]. In both studies, tumors with these fusions mostly showed overlapping features between leiomyoma and LG-ESS, but a few tumors with high-grade features were also present [9, 11]. In the first study, the authors described 13 cases, of which 10 were originally diagnosed as LG-ESS, and one case each was diagnosed as LMS, leiomyoma, and undifferentiated uterine sarcoma [9]. The reported assigned diagnoses have also changed over time during the diagnostic process, based on molecular findings and observations in the recurrent tumors, and included also uterine tumor resembling ovarian sex cord tumor, cellular leiomyoma, and myxoid leiomyosarcoma. Apart from one tumor with high-grade features, all other tumors showed bland morphology and were well circumscribed in most cases, but despite this commonly behaved in malignant fashion. Three patients died of the disease, three were alive with disease, five showed no evidence of disease, and in two recent cases, the follow-up was not available. In the second study on 16 tumors from 12 patients, the initial diagnosis was leiomyoma in 6/16 tumors, ESN or endometrial stromal tumor in 4/16 cases, LG-ESS in 4/16 cases, and intravenous leiomyomatosis in 2/16 cases [11]. The follow-up was available for 9 patients, of whom 3 relapsed and 6 were without evidence of disease. In our study, 5/8 patients showed recurrent disease, two of which died of disease and three were alive with disease.

The differential diagnosis of sarcomas with *KAT6B/A::KANSL1* fusion showing low-grade features mainly includes tumors with endometrial stromal or smooth muscle differentiation. Most cases in the published studies were originally diagnosed as LG-ESS, but given the sharp demarcation of some tumors, they can also be misdiagnosed as endometrial stromal nodules. According to current knowledge, reliably rendering the correct diagnosis based only on morphological and immunohistochemical features is not possible. The morphology of sarcomas with *KAT6B/A::KANSL1* fusion is variable, but almost all cases exhibited some features suggestive of endometrial stromal differentiation (fibroblastic or fibromyxoid) or hybrid features between endometrial stromal and smooth muscle differentiation. Rarely, only features suggestive of smooth muscle differentiation can be found. Moreover, rare tumors can show high-grade features (6% of all reported cases, including ours), and these cannot be differentiated from high-grade endometrial stromal sarcoma or undifferentiated uterine sarcoma based on morphological features alone. Concerning the morphological features of tumors with low-grade appearance, the cells can be ovoid, spindled, or both, with usually round or oval, and rarely spindle-shaped nuclei. Mitotic activity is variable and can exceed 20 mitoses/10 HPF, but mostly is up to 5/10 HPF. Nuclear atypia is mild or moderate. The architecture is typically heterogeneous, including solid, nested, fascicular, and storiform patterns, and in some cases even schwannoma-like or sex cord-like features. The whorling pattern which is characteristic for fibromyxoid LG-ESS is commonly present, as are hyaline collagen deposits. The vasculature typically consists of arterioliform vessels typical for endometrial stromal tumors, but larger, thick-walled vessels are also commonly found. Pericytoma-like vasculature has been reported as a common finding, but in our study, it was present in only two cases. The demarcation from the surrounding myometrium is typically sharp, but the infiltrative pattern and tongue-like invasion characteristic of LG-ESS can also be seen.

Immunohistochemically, the combined data from our and both previously published studies suggest that most tumors express estrogen (91%) and progesterone receptors (89%) and show variable expression of smooth muscle and “endometrial stromal” markers [9, 11]. The expression of smooth muscle markers seems to be limited in both the spectrum of markers expressed and their extent of positivity. The most commonly expressed marker was smooth muscle actin (88%). The expression of transgelin and calponin was found in 56% cases, desmin in 39% cases, and caldesmon in 25% cases. The tumors showed common expression of endometrial stromal markers, such as CD10 (92%) and IFITM1 (73%). Nevertheless, these markers are not specific and CD10 especially is typically expressed not only in LG-ESS/ESN, but also in a subset of leiomyomas, especially the cellular variant [25]. The expression of WT1 seems to be at most focal and weak in most sarcomas with *KAT6B/A::KANSL1* fusion, and 50% of the analyzed cases were entirely negative, which would be unusual for LG-ESS. According to current knowledge, sarcomas with *KAT6B/A::KANSL1* fusion cannot be diagnosed based only on morphological and immunohistochemical features. Tumors with features suggestive of this diagnosis, particularly including overlapping features between endometrial stromal and smooth-muscle differentiation, should undergo molecular testing, preferably by NGS, which also allows the assessment of other recurrent molecular alterations typical for some other entities entering the differential diagnosis. Alternatively, our study showed that the *KAT6B/A::KANSL1* fusion can be reliably detected by FISH analysis using an appropriate pair of fusion probes.

One of the previous studies proposed a diagnostic algorithm for these tumors, incorporating clinical features (perimenopausal women and uterine corpus location), microscopic and immunohistochemical features, and molecular findings [11]. Concerning microscopy, the authors emphasized some relevant features such as the presence of hybrid morphology of leiomyoma and LG-ESS or ESN, whorling pattern, arterioliform vasculature, central hyalinized vessels, and spindled or ovoid cells with mild to moderate atypia and few mitoses. Immunohistochemically, the tumors are supposed to be characterized by estrogen and progesterone receptor expression, and variable expression of smooth muscle markers and endometrial stromal markers, such as CD10 and IFITM1. However, applying both the clinical and pathological features, our cases would not entirely fit based on this suggested algorithm.

The potential additional molecular alterations beyond *KAT6/B::KANSL1* gene fusion in these tumors have not been widely investigated. In one previous study, the authors did not detect any pathogenic variants in their cohort [11]. However, this might be attributed to the imperfect method used in this study for generating those results, as their mutation analysis was conducted using cDNA (synthesized from RNA). This approach is not optimal for mutation detection, especially for FFPE samples, as it often results in insufficient coverage, particularly for genes with low transcript levels [26]. In another recent study, the authors focused on methylation profiling and copy number alterations in 18 sarcomas with *KAT6B/A::KANSL1* fusion [10]. Their results showed that aggressive tumors have a different methylation profile, but also harbor various copy number alterations, including deletions of the *CDKN2A/B* and *NF1* loci. In our study, we detected additional pathogenic or likely pathogenic mutations in all 5 aggressive tumors, including mutations in *FANCD2, RB1, PTEN, ATM, TP53, NF1, TSC1, CREBBP,* or *PDGFRB* genes*.* All these genes are involved in complex signaling pathways that regulate DNA repair, cell cycle control, and cell proliferation. Moreover, one patient without evidence of disease had a mutation in *ERCC3* gene, which is involved in DNA damage repair, and one patient also with no evidence of disease had no detected mutations. All the detected mutations can also be observed in tumors with smooth muscle and endometrial stromal differentiation and are not specific for tumors with the *KAT6B/A::KANSL1* fusion. The mutation landscape varies within the group of sarcomas with the *KAT6B/A::KANSL1* fusion, as does the mRNA expression within this group (PCA analysis) and both might be linked to differences in clinical behavior. Our results, together with the results of previously published work, suggest that both the presence of additional molecular alterations (copy number variations or mutations) or the differences in methylation profiling may be used as a surrogate marker in the identification of tumors with aggressive behavior.

Transcriptome analysis comparing the expression profile of sarcoma with *KAT6B/A::KANSL1* fusion versus LG-ESS tumors revealed an upregulation of genes primarily involved in DNA replication, cell cycle regulation, and chromatin modification, and a downregulation of several proteasome subunits in the *KAT6B/A::KANSL1* group. These differences further support the notion that sarcomas harboring *KAT6B/A::KANSL1* fusions are in fact a distinct entity from LG-ESS, as was also suggested in the recent study based on methylation profiles [10]. However, further research is needed to clarify the underlying mechanisms driving these differences.

We are aware of the limitations of our study, which are mainly related to the small number of cases, which makes the comparison between cases with benign and aggressive behavior difficult and impacts the statistical significance of our findings.

In conclusion, the results of our study and previous research suggest that, despite some overlapping morphological and immunohistochemical features with tumors of smooth muscle and endometrial stromal differentiation (LG-ESS and ESN), sarcomas with the *KAT6B/A::KANSL1* fusion represent a distinct entity rather than merely a subtype of LG-ESS. It is important to be aware of this entity since despite the commonly bland morphology, the tumors have a propensity for aggressive behavior. Given the sharp circumscription of some tumors, misdiagnosing them as benign lesions, such as endometrial stromal nodule or leiomyoma, can be particularly problematic. Our results suggest that the molecular profile of tumors with aggressive behavior is different from those with benign clinical course, including additional mutations in aggressive cases and differences in mRNA expression. However, further investigation on a larger cohort of cases is needed to confirm these findings.

## Supplementary Information

Below is the link to the electronic supplementary material.

Supplementary file1 (DOCX 478 KB)

Supplementary file2 (DOCX 403 KB)

Supplementary file3 (DOCX 426 KB)

Supplementary file4 (DOCX 122 KB)

Supplementary file5 (DOCX 284 KB)

Supplementary file6 (XLSX 12 KB)

Supplementary file7 (XLSX 46 KB)

Supplementary file8 (XLSX 13 KB)

Supplementary file9 (XLSX 13 KB)

Supplementary file10 (XLSX 11 KB)

Supplementary file11 (XLSX 692 KB)

Supplementary file12 (XLSX 300 KB)

## Data Availability

The datasets used and/or analyzed during the current study are available from the corresponding author upon reasonable request.
