# Supplementary material for: Uterine sarcoma with KAT6B/A::KANSL1 fusion: a molecular and clinicopathological study on 9 cases
Source: Virchows Arch. 2024 Dec 4;486(3):551–62. doi: 10.1007/s00428-024-03994-3 (PMC11950137; doi:10.1007/s00428-024-03994-3)
Supplement: Supplementary file 1 — Supplementary file1 (DOCX 478 KB) [file 428_2024_3994_MOESM1_ESM.docx]

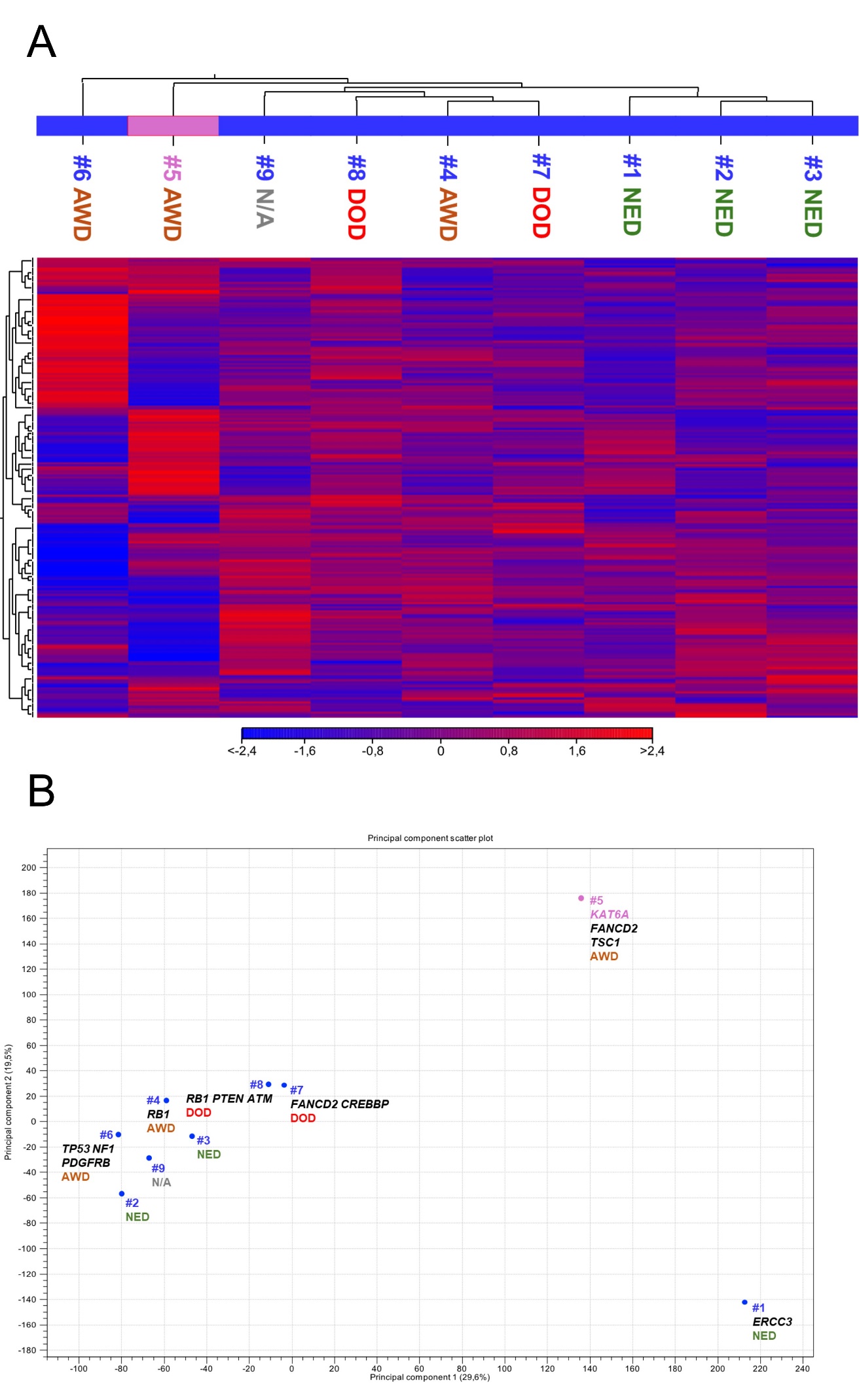


**Supplementary Figure 1:** A Unsupervised hierarchical clustering analysis of 9 *KAT6A/B::KANSL1* samples. The heatmap illustrates the clustering of both samples and genes (10 thousand features with a minimum of 5 thousand counts in at least one sample) based on expression similarities. Samples are represented along the horizontal axis, while genes are arranged vertically. The dendrogram at the top of the heatmap depicts the hierarchical relationships among samples, with shorter branches indicating greater similarity. Gene expression levels are represented by a color gradient, with red indicating high expression and blue denoting low expression. B A Principal Component Analysis (PCA) plot depicting the first and second principal components. AWD: alive with disease; DOD: died of disease; NED: no evidence of disease; NA: not available
