# Supplementary material for: Uterine sarcoma with KAT6B/A::KANSL1 fusion: a molecular and clinicopathological study on 9 cases
Source: Virchows Arch. 2024 Dec 4;486(3):551–62. doi: 10.1007/s00428-024-03994-3 (PMC11950137; doi:10.1007/s00428-024-03994-3)
Supplement: Supplementary file 2 — Supplementary file2 (DOCX 403 KB) [file 428_2024_3994_MOESM2_ESM.docx]

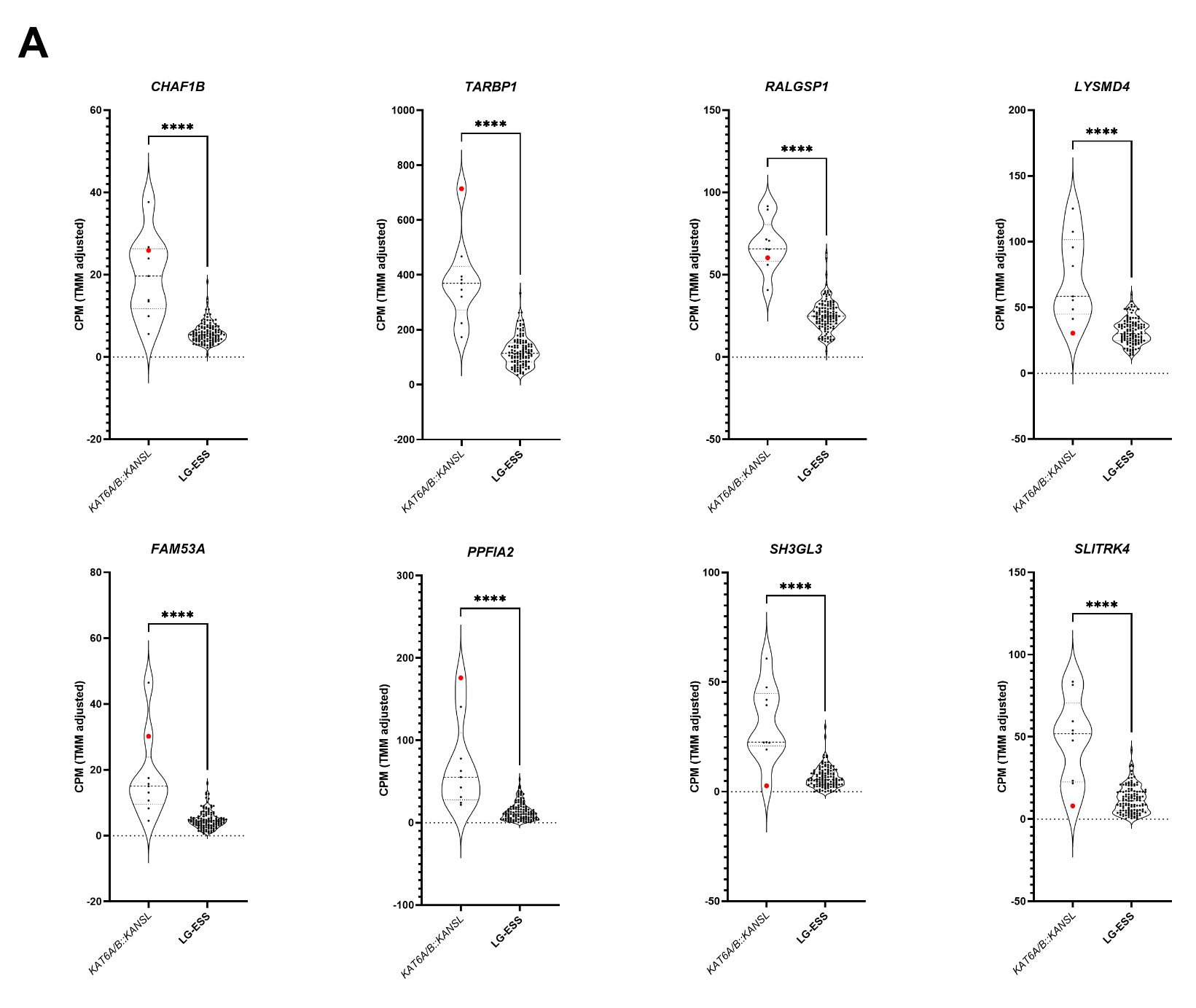

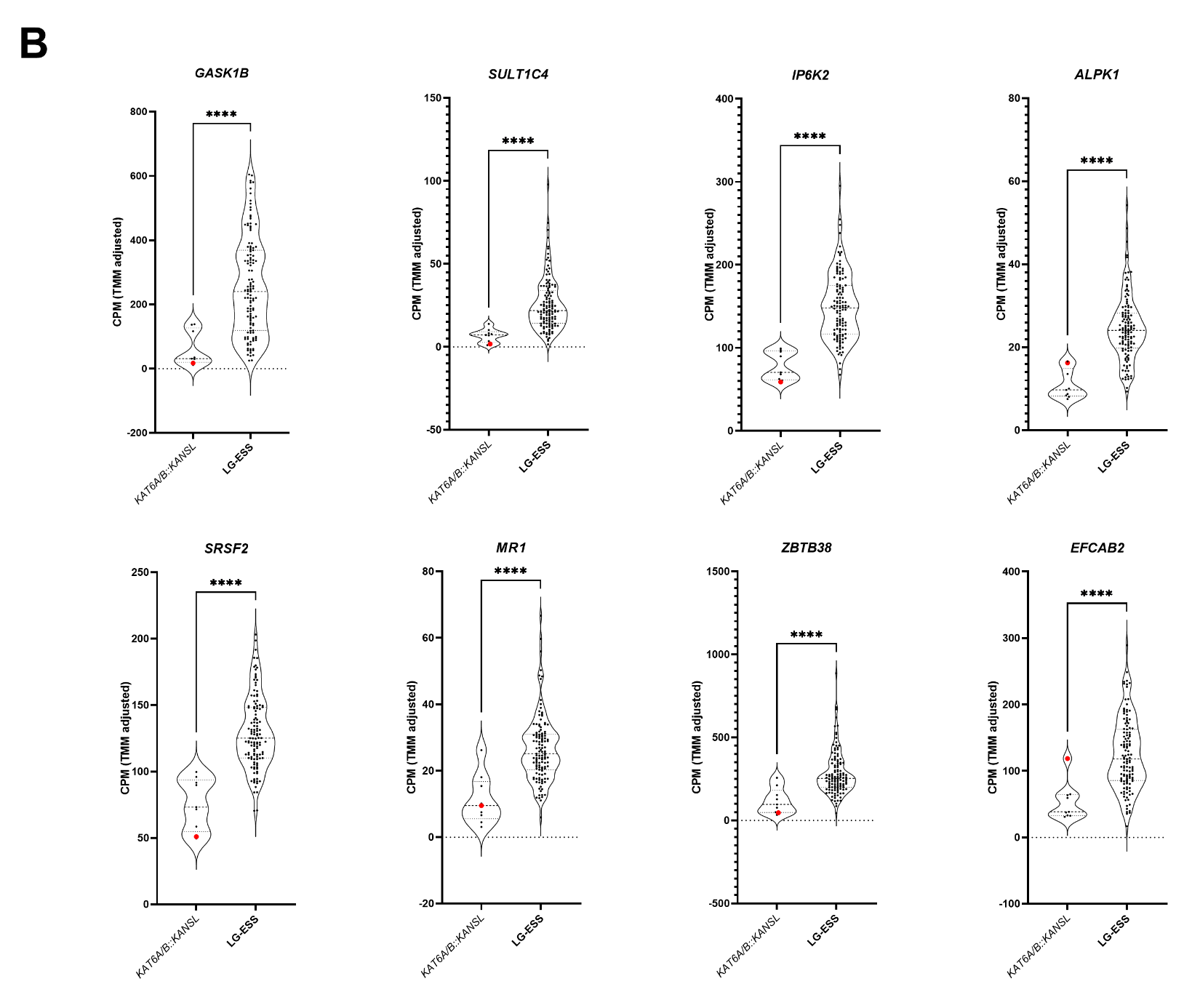


**Supplementary Figure 2:** Differential gene expression between the *KAT6A/B::KANSL1* and LG-ESS cohorts. Violin plots depicting the eight most uniformly expressed genes upregulated in the *KAT6A/B::KANSL1* cohort compared to the LG-ESS cohort (A) and the eight most uniformly expressed genes downregulated in the *KAT6A/B::KANSL1* cohort compared to the LG-ESS cohort (B). The y-axis represents the CPM (TMM-adjusted) values for each gene. The x-axis shows the two cohorts: *KAT6A/B::KANSL1* (n=9) and LG-ESS (n=123). Case #6 is highlighted in the plots (red dot). A Mann-Whitney U test was performed to identify significantly differentially expressed genes between the two groups (p < 0.0001).
