# Supplementary material for: Uterine sarcoma with KAT6B/A::KANSL1 fusion: a molecular and clinicopathological study on 9 cases
Source: Virchows Arch. 2024 Dec 4;486(3):551–62. doi: 10.1007/s00428-024-03994-3 (PMC11950137; doi:10.1007/s00428-024-03994-3)
Supplement: Supplementary file 3 — Supplementary file3 (DOCX 426 KB) [file 428_2024_3994_MOESM3_ESM.docx]

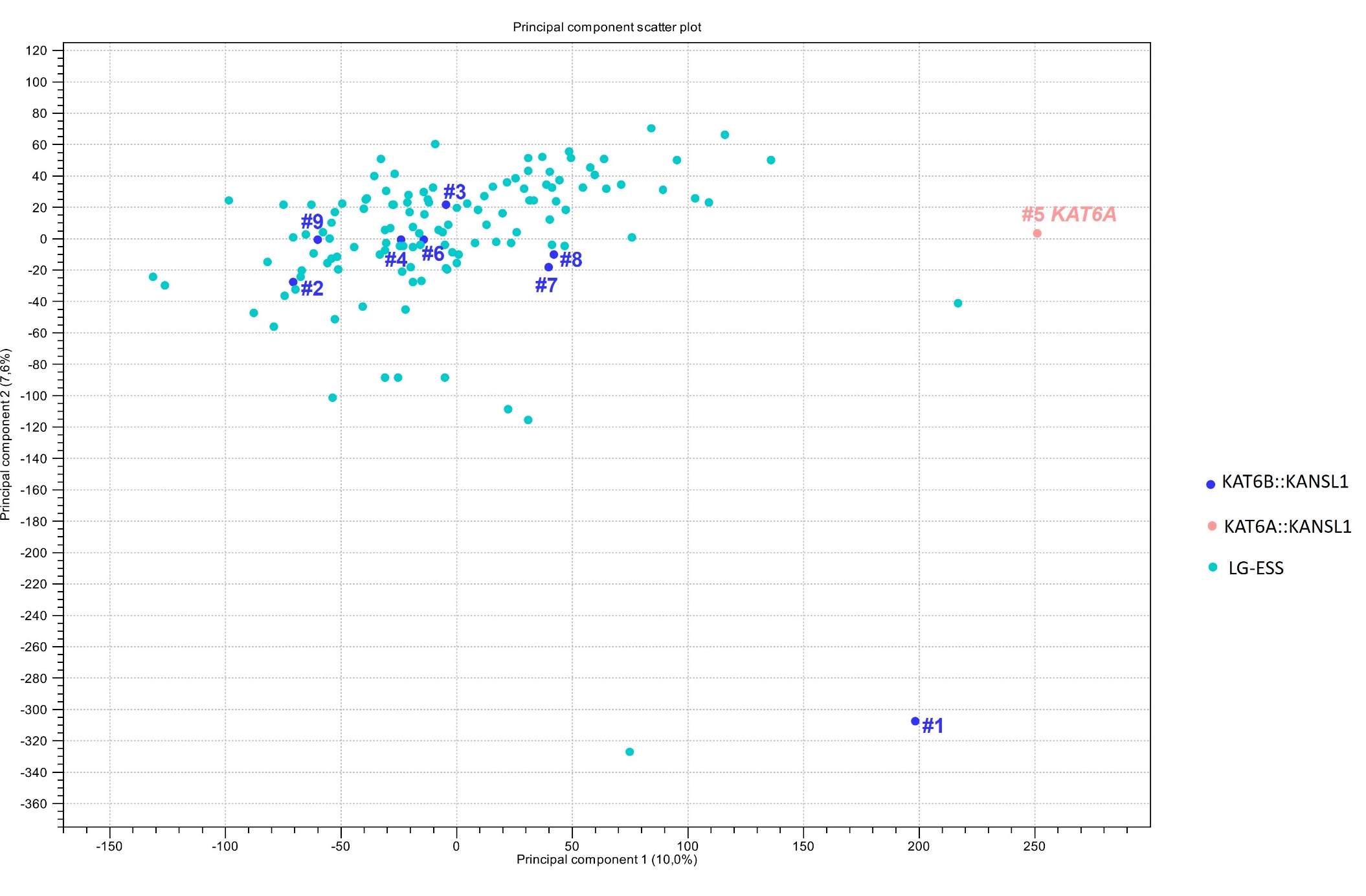


**Supplementary Figure 3:** Principal Component Analysis (PCA) comparing molecular profiles of sarcomas harboring KAT6B/A::KANSL1 fusion genes versus LG-ESS. Sample numbers indicate individual cases within the KAT6B/A::KANSL1 fusion-positive sarcoma cohort.
