# Supplementary material for: Uterine sarcoma with KAT6B/A::KANSL1 fusion: a molecular and clinicopathological study on 9 cases
Source: Virchows Arch. 2024 Dec 4;486(3):551–62. doi: 10.1007/s00428-024-03994-3 (PMC11950137; doi:10.1007/s00428-024-03994-3)
Supplement: Supplementary file 4 — Supplementary file4 (DOCX 122 KB) [file 428_2024_3994_MOESM4_ESM.docx]

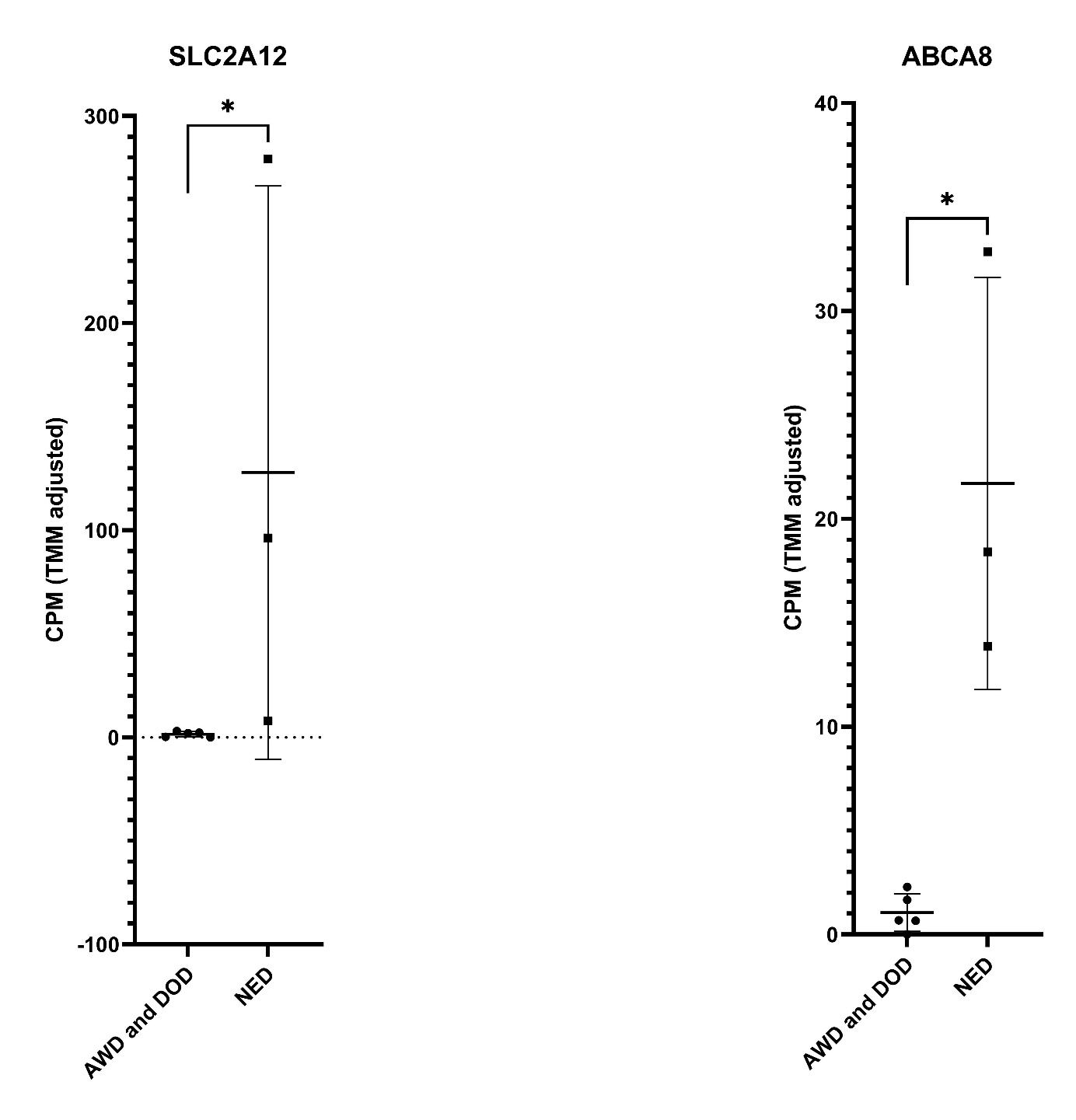


**Supplementary Figure 4:** Differential gene expression in patients with KAT6A/B::KANSL1 fusion based on clinical outcome.

Gene expression levels of SLC2A12 and ABCA8 were compared between two cohorts of patients harbouring KAT6A/B::KANSL1 fusion. The cohorts were defined by clinical outcome: AWD/DOD (alive with disease/died of disease, n=4) and NED (no evidence of disease, n=3). The AWD/DOD group comprised samples #4, #5, #6, #7, and #8, while the NED group included samples #1, #2, and #3. Gene expression is represented as CPM (counts per million) values, normalized using the trimmed mean of M-values (TMM) method. Each point represents an individual sample, with horizontal lines indicating group means. Statistical significance between groups was assessed using the Mann-Whitney U test, with p < 0.05 considered statistically significant.
