# Supplementary material for: Uterine sarcoma with KAT6B/A::KANSL1 fusion: a molecular and clinicopathological study on 9 cases
Source: Virchows Arch. 2024 Dec 4;486(3):551–62. doi: 10.1007/s00428-024-03994-3 (PMC11950137; doi:10.1007/s00428-024-03994-3)
Supplement: Supplementary file 5 — Supplementary file5 (DOCX 284 KB) [file 428_2024_3994_MOESM5_ESM.docx]

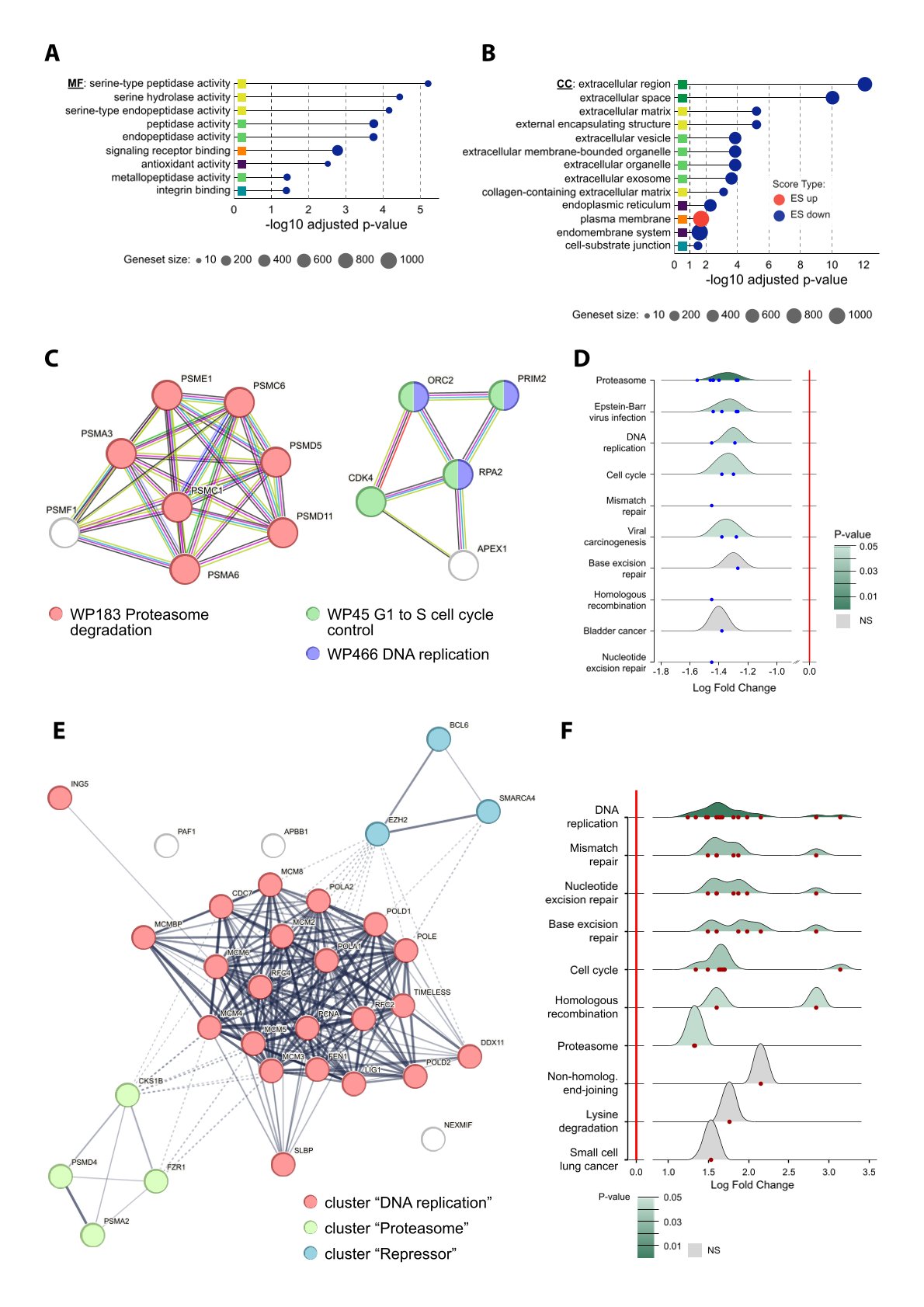


**Supplementary Figure 5:** Gene set enrichment analyses of genes differentially expressed in *KAT6A/B::KANSL1* tumors when compared to LG-ESS. A) and B) Gene set Ordinal Association Test (GOAT) was used to identify enriched categories within the GO database domain Molecular Functions (MF; A) and Cellular Component (CC; B). Effect size (ES) score type "ES up" (red circles) indicates that the gene set is enriched in genes with a positive gene effect size, "ES down" (in dark blue) indicates enrichment of negative gene effect sizes. Colored squares indicate the hierarchical clustering of the gene sets. C-F Categories of genes downregulated (C and D) or upregulated (E and F) in *KAT6A/B::KANSL1* tumors. C. Functional protein association network (STRING) of the genes displaying reduced levels in *KAT6A/B::KANSL1* tumors. The most significantly affected WikiPathways are highlighted in colors. D. GSEA results visualized as a ridgeline plot, with overrepresentation analysis performed against the category of KEGG Pathways. E and F represent the results of the same analyses as in C and D, performed within the list of genes significantly upregulated in *KAT6A/B::KANSL1* tumors.
